# Relationship of SOD-1 Activity in Metabolic Syndrome and/or Frailty in Elderly Individuals

**DOI:** 10.3390/metabo14090514

**Published:** 2024-09-23

**Authors:** Sylwia Dzięgielewska-Gęsiak, Ewa Wysocka, Edyta Fatyga, Małgorzata Muc-Wierzgoń

**Affiliations:** 1 Department of Internal Diseases Propaedeutics and Emergency Medicine, Faculty of Public Health in Bytom, Medical University of Silesia in Katowice, Piekarska 18 Str., 44-902 Bytom, Poland; efatyga@sum.edu.pl (E.F.); mwierzgon@sum.edu.pl (M.M.-W.); 2 Department of Laboratory Diagnostics, Poznan University of Medical Sciences, 84 Szamarzewskiego Str., 60-569 Poznań, Poland; ewysocka@ump.edu.pl

**Keywords:** aging, metabolic syndrome, frailty syndrome, SOD-1

## Abstract

Introduction: Although aging is a natural phenomenon, in recent years it has accelerated. One key factor implicated in the aging process is oxidative stress. Oxidative stress also plays a role in frailty (frail) and metabolic syndrome (MetS). Methods: A total of 66 elderly persons (65 years old and older) with no acute or severe chronic disorders were assessed for waist circumference (WC), arterial blood pressure, glycemia, glycated hemoglobin (HbA1c), plasma lipids, and activity of erythrocyte superoxide dismutase (SOD-1). Patients were classified as NonMetS-Nonfrail (n = 19), NonMetS-frail (n = 20), MetS-Nonfrail (n = 17), or MetS-frail (n = 10). Results: There were no significant differences in superoxide dismutase activity among investigated elderly groups. However, the data suggest that MetS individuals, both frail and nonfrail, have higher risk factors for cardiovascular disease compared to NonMetS individuals. The correlations analyses of SOD-1 and other metabolic indices suggest that SOD-1 levels may be influenced by age, total cholesterol, HDL cholesterol, and fasting glucose levels in certain groups of seniors. Conclusions: Aging is associated with decreased antioxidant enzyme SOD-1 activity with glucose alteration in frailty syndrome as well as with lipids disturbances in metabolic syndrome. These factors provide a nuanced view of how frailty and metabolic syndrome interact with various health parameters, informing both clinical practice and future research directions.

## 1. Introduction

Due to the increasing human lifespan, advancements in civilization, and improvements in the quality of life worldwide, the percentage of people in the post-productive age group is steadily rising [1]. Although aging is a natural phenomenon, in recent years it has accelerated significantly compared to the birth rate and the number of people in the working age group [2]. The percentage of elderly individuals globally is projected to double by 2050, with developed countries like Japan and the EU showing high numbers [3,4,5]. In Poland, the proportion of the working age group is projected to decrease by 2050, with implications on healthcare services [6].

The aging process of the body contributes to an increase in health issues, chronic diseases, and disabilities. Studies on the health status of individuals aged 60 and above reveal that 50% of them suffer from three or more metabolic disorders and chronic diseases, forming a unique health profile for each patient [7,8,9,10]. Common disorders include hyperglycemia, dyslipidemia, and hypertension, which characterize metabolic syndrome [11]. However, disease symptoms in old age often present atypically and nonspecifically, making early detection and treatment challenging [12]. Age-related health problems, alongside a decline in bodily reserves, can lead to multiple organ dysfunctions. These dysfunctions result in functional changes that reduce the operational capacities of all organs and systems, giving rise to geriatric syndromes such as frailty syndrome, heightened fall risks, increased mortality rates, nutrition disorders, cognitive impairments, and depression [13,14,15].

Frailty syndrome is a clinical description of multisystem changes related to the aging process and is often mistakenly equated with multimorbidity or disability, which may only be an underlying cause. The syndrome is described as a complex, multidimensional state marked by a reduced physiological reserve and decreased resilience to stressors due to diminished organ and system functioning [16,17].

Despite that aging is a natural process characterized by a gradual decline in physiological functions (taste and smell decrease, while fat mas tends to increase) the process increases susceptibility to diseases. One key factor implicated in the aging process is oxidative stress, resulting from an imbalance between reactive oxygen species (ROS) production and the body’s detoxification ability [17,18].

Reactive oxygen species (ROS) and free radicals are important components of cellular metabolism, playing roles in both normal physiological functions and the pathogenesis of various diseases. The following are the primary sources of ROS and free radicals:

- Mitochondrial Electron Transport Chain: Within mitochondria, during cellular respiration, the oxidative phosphorylation involves electron transfer that can produce ROS such as superoxide anion (O2^•−^) and hydrogen peroxide (H_2_O_2_). Imperfections in the electron transport system may increase ROS production.
- Reactions Catalyzed by Oxidoreductases: Oxidoreductase enzymes participate in redox reactions during metabolic processes, such as lipid peroxidation, which can inadvertently produce ROS as metabolic byproducts (resulting in the formation of reactive aldehydes), playing a role in regular cellular metabolism.
- Oxidation of Low-Molecular-Weight Compounds (RH2): Various compounds such as amino acids, thiols, and reducing sugars can undergo oxidation, leading to the production of ROS (e.g., protein oxidation), including superoxide anions and free radicals derived from oxidized compounds (•RH).
- Peroxisomes: Organelles that contain enzymes, such as xanthine oxidase, are responsible for purine metabolism and the oxidation of fatty acids, which also generates ROS.
- Phagocyte Activation: Neutrophils and other phagocytic cells produce ROS, such as superoxide anion and hydrogen peroxide, in response to infections via NADPH oxidase. This process is crucial for their bactericidal activity, and the Fenton reaction (production of hydroxyl radical in the presence of Fe^2+^ ions) can further increase the amount of ROS [19,20].

All these sources generate ROS that are crucial for various biological processes, but their excess can lead to oxidative stress and cellular damage. Therefore, regulation of reactive oxygen species levels is crucial for maintaining balance in the organism. What is more, reports have supported the role of oxidative stress in the development of aging processes in human organism [21].

The first line of protection features enzymatic antioxidants that transform already existing ROS into less harmful entities, along with proteins that restrict the availability of transition metal ions for Fenton reactions, thereby preventing the generation of additional ROS. Reviews have revealed interesting links between antioxidant enzymatic defense capacity and aging, showing a decrease in the crucial activity of antioxidative enzymes with age [22]. The enzyme, such as superoxide dismutase 1 (SOD-1), is fundamental component of the intracellular defense system. Superoxide dismutase 1 (SOD-1) plays a crucial role in protecting cells from oxidative stress by converting superoxide radicals into oxygen and hydrogen peroxide. SOD-1 regulates the levels of superoxide anion in the cytosol, which is essential for normal cellular metabolism, including the regulation of signaling pathways and the defense against pathogens. However, its levels can also be affected by diseases and disturbed metabolic processes as well as external factors, thereby limiting the possibility of potential formation of hydroxyl radicals from •O^2−^. The SOD-1 enzyme is a homodimer with a molecular weight of 32 kDa [23]. The protein part (apoenzyme) is synthesized on ribosomes in the form of monomers. Transcription factors that play a role in regulating gene expression for SOD-1, whether constitutive or induced, include nuclear factor kappa B (NF-κB), which is sensitive to changes in the redox state; SP1 and the AP-1 protein complex, which respond to cytokines and oxidative stress; the AP-2 protein family, which can enhance sod1 transcription; and CCAAT-binding proteins (C/EBP) that bind to regulatory and enhancer sequences [24]. The stability of SOD-1 relies on zinc (Zn^2+^) ions incorporated into the apoenzyme, which help maintain its structural integrity, and copper (Cu^2+^) ions, which are essential for its catalytic (oxidoreductive) function [23]. Inhibition or acceleration of the enzyme’s process in the cytoplasm is the element of the post-translational modification of SOD-1. The end effect, which refers to the enzyme’s activity, is not merely a direct representation of the gene structure in terms of the quantity of enzymatic protein and its function.

The clinical significance of superoxide dismutase (SOD-1) was initiated by neurology. Amyotrophic lateral sclerosis (ALS), particularly the familial form, is a condition in which the activity of superoxide dismutase has been firstly and most thoroughly studied [25,26].

A study by Kozakiewicz et al. has suggested that the decrease in SOD-1 activity may contribute to the aging process by increasing the accumulation of ROS and causing oxidative damage to cells and tissues [27].

The imbalance between pro-oxidative processes (i.e., lipid peroxidation, hyperglycemia) and the capabilities of antioxidant defense also play a significant role in conditions such as diabetes, cardiovascular diseases, chronic inflammation conditions, cancer, and anemia [28,29,30]. A study conducted on patients like those with metabolic syndrome or ischemic heart disease has allowed us to consider oxidative stress as a connecting element linking various directions of metabolic disturbances in the aforementioned lifestyle diseases [31]. Additionally, research has shown that SOD-1 activity is reduced in frail individuals, which may contribute to the aging process and the development of age-related diseases [32,33]. This can lead to the development of age-related diseases such as neurodegenerative disorders, cancers, and cardiovascular diseases. Furthermore, changes in SOD-1 activity have been linked to metabolic syndrome—the metabolic disorders characterized by obesity, hypertension, hyperglycemia, and dyslipidemia [34]. This suggests that impaired SOD-1 function may also play a role in the pathogenesis of age-related diseases.

Thus, we have studied the activity of erythrocyte superoxide dismutase-1 (SOD-1) with regard to clinical conditions: metabolic syndrome and/or frailty syndrome diagnosed in elderly population. 

## 2. Materials and Methods

The study was performed in accordance with the Declaration of Helsinki of 1975 for Human Research revised in 2013. The Bioethics Committee of Medical University of Silesia in Katowice (statement number: PCN/0022/KB1/75/I/20/21) approved the study protocol; all participants signed consent, after they had been informed.

### 2.1. Inclusion Criteria

A total of 541 elderly Caucasian individuals aged ≥65 years old who did not have any diagnosed acute and/or chronic disorders, were not taking medication, and were not on any special diets or supplements were invited to the study.

### 2.2. Exclusion Criteria

Individuals with a history of certain medical conditions such as cardiovascular diseases, diabetes, cancers, inflammatory disorders, liver failure, and impaired kidney function (eGFR lower than 60 mL/min/1.73 m^2^), as well as those with severe anemia (based on CBC criteria), were not part of the study. Additionally, participants who were smokers and or alcohol drinkers (former or current) were also excluded from the study. Furthermore, those elderly participants who were taking any medications such as those for elevated blood pressure, hypertriglicerydemia, and low HDL cholesterol; antihyperglycemic drugs; or antioxidants (including OTC vitamins and supplements) were excluded.

### 2.3. Clinical Examination

Following a medical history, a physical examination was performed, which included measuring waist circumference (WC) at the smallest girth around the navel using a non-elastic tape (recorded to the nearest 0.1 cm) and blood pressure (BP). The systolic (SBP) and diastolic (DBP) blood pressure were measured as per the guidelines of the European Society of Hypertension [35]. The average of blood pressure readings was calculated and utilized in all the analyses.

### 2.4. Frailty Diagnosis

The Clinical Frailty Scale (CFS) was used to determine if elderly patients were or were not frail [36] The components of this syndrome that were of authors’ interests included unintentional weight loss, strength reduction, slowness (walking speed reduction), low physical activity, and fatigue were of authors interests. Frail patients were considered frail older people who scored for three or more components, and nonfrail patients were those who score two or less of the components described.

### 2.5. Blood Sampling and Biochemical Analysis

The test samples, including total blood, serum, and heparin plasma, were obtained from patients following the current guidelines for patient preparation and sample collection. Finally, 66 individuals were investigated fasting blood samples. The samples were collected into vacutainer tubes in the morning after at least 12 h of fasting.

For each patient, a complete blood count (CBC) was obtained by hematological analyzer Sysmex 4500 (Siemens, Los Angeles, CA, USA).

#### 2.5.1. Glucose and Lipids Measurements

The concentrations of glucose, total cholesterol (T-C), HDL cholesterol (HDL-C), LDL cholesterol (LDL-C), and triglycerides (TG) were measured in fresh blood samples on the automatic biochemistry analyzer Dimension EXL (Siemens Healthcare Diagnostics Inc., Tarrytown, NY, USA) with enzymatic methods. Low-density lipoproteins (LDL) were calculated using the Friedewald formula.

#### 2.5.2. The Activity of Cu-, Zn-Superoxide Dismutase (SOD-1) in Red Blood Cell

The method uses xanthine and xanthine oxidase to produce superoxide radicals, which then interact with 2-(4-iodophenyl)-3-(4-nitrophenol)-5-phenyltetrazolium chloride (INT) to create a red formazan dye. The activity of SOD-1 is determined by measuring the extent of inhibition in this reaction, with kinetics measured at 505 nm. The intra-assay and inter-assay coefficients of variation for SOD-1 were 2.0% and 3.9%, respectively.

##### Preparation of Hemolysate

First, 0.5 mL of whole heparinized blood was centrifuged at 3000 rpm for 10 min. The plasma was discarded, leaving the leukocyte-platelet buffy coat. Then, 3 mL of 0.9% NaCl was added to the red blood cells, followed by another centrifugation for 10 min at the same speed, and the supernatant was removed. This washing step was repeated four times. The washed red blood cells were then put in cold redistilled water at a volume of 2 mL and incubated at +4 °C for 15 min to obtain the hemolysate. In another tube, 5.0 mL of 0.01 M phosphate buffer (pH 7.0) was prepared; 0.2 mL was removed, and 0.2 mL of the hemolysate was added, resulting in a 25-fold dilution for measurement.

##### Measurement Procedure

The procedure was carried out at 37 °C. To 0.05 mL of hemolysate and 0.01 M phosphate buffer (blank) along with diluted standards, 1.7 mL of a mixture (0.05 mM xanthine, 0.025 mM I.N.T., 0.94 mM EDTA, and 50 mM CAPS at pH 10.2) was added and mixed. Next, 0.25 mL of xanthine oxidase solution (80 U/L) was added, and after mixing, initial absorbance (A1) was measured after 30 s, followed by final absorbance (A2) after 180 s to calculate ΔA/min. Measurements were taken at 505 nm in a 1 cm cuvette.

##### Calculating SOD-1 Activity

The ΔAO/min of the blank (100%) represents the unimpeded reaction. The percentage of inhibition for the test sample was calculated using the following formula:

% inhibition = ΔAB/min × 100%/ΔAO/min

A relationship between % inhibition and log10 [SOD-1 U/mL] was plotted, resulting in a linear equation y = ax + b. The ΔAB/min of the test sample was then used to determine % inhibition, leading to x (log10 [SOD-1 U/mL]), which was converted to SOD-1 activity in U/mL of hemolysate. After adjusting for hemoglobin concentration, SOD-1 activity was expressed in U/g Hb. Quality control was conducted with RANSOD Control from RANDOX, using reagents from Randox Laboratories and a StatfaxTM 1904 Plus spectrophotometer (Awareness Technology, Inc., Palm City, FL, USA).

### 2.6. Metabolic Syndrome

Metabolic syndrome was identified as recommended by the NCEP ATPIII [37], which considers the combination of at least three components: abdominal obesity (measured by waist circumference, >102 cm for men and >88 cm for women); blood pressure ≥ 130 mmHg (systolic) or ≥85 mmHg (diastolic); triglycerides ≥ 150 mg/dL; HDL cholesterol < 40 mg/dL for men and <50 mg/dL for women; and fasting glucose ≥ 100 mg/dL. The presence of three out of five abnormal findings constituted a diagnosis of the MetS and allowed to divide groups into MetS and NonMetS.

HDL cholesterol levels, to protect against atherosclerosis, should be >40 mg/dL for men and >50 mg/dL for women.

### 2.7. Group Analysed in the Study

Based on metabolic syndrome diagnosis and frailty scale, finally, four groups were considered: NonMetS-Nonfrail (n = 19), NonMetS-frail (n = 20), MetS-Nonfrail (n = 17), and MetS-frail (n = 10).

### 2.8. Statistical Analysis

Statistical analysis was calculated by Statistica (version 13.3) for Windows. The Shapiro–Wilk test was used to check the normality of distributions of variables in the 66 elderly individuals (which means 65 years old or older) and metabolic syndrome and frailty syndrome elderly groups. Because most of the data had a non-normal distribution, data were shown as medians and lower and upper quartiles, and a nonparametric Kruskal–Wallis test with post hoc analysis of multiple comparisons was done. Correlations between the studied variables were assessed using Spearman’s R coefficient. The degree of association between variables was categorized according to the correlation coefficient values as follows: negligible for R < 0.0, weak for R between 0.1 and <0.3, moderate for R between 0.3 and <0.5, strong for R between 0.5 and <0.7, very strong for R between 0.7 and <0.9, and nearly perfect for R between 0.9 and 1.0. *p* value of less than 0.05 was considered as significant.

## 3. Results

The data provided in Table 1 are a comparison of various clinical and biochemical parameters among different elderly groups of individuals categorized as NonMetS- Nonfrail (0), NonMetS-frail (1), MetS-Nonfrail (2), and MetS-frail (3). 

**Figure 1:**
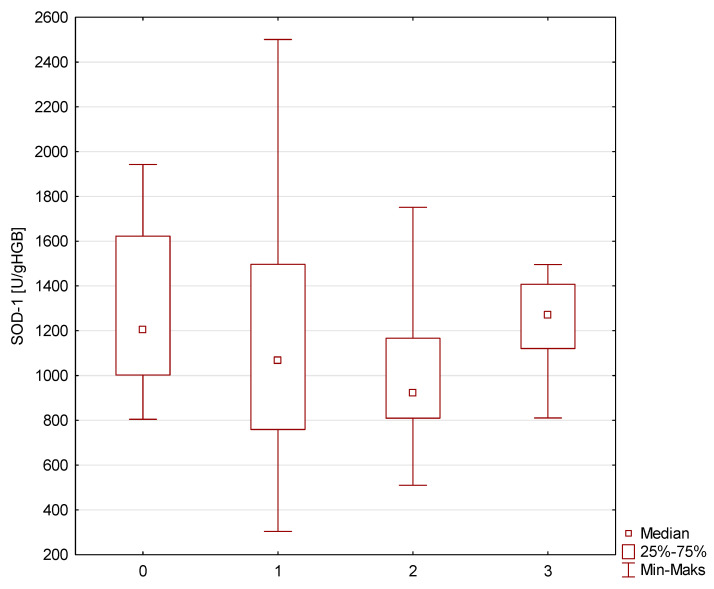
SOD-1 concentration in investigated group (NonMetS-Nonfrail (0), NonMetS-frail (1), MetS-Nonfrail (2), and MetS-frail (3)).

There were no significant differences in diastolic blood pressure, mean glucose concentration, glycated hemoglobin levels, and SOD-1 activity among investigated groups. Additionally, we added Figure 1 with SOD-1 activity in all compared groups (NonMetS- Nonfrail (0), NonMetS-frail (1), MetS-Nonfrail (2), and MetS-frail (3)).

MetS-Nonfrail elderly individuals had a significantly higher waist circumference, systolic blood pressure, and fasting glucose concentration and a significantly worse lipid profile compared to the other groups. However, post hoc analyses showed only significant results in MetS-Nonfrail group for waist circumference (MetS-Nonfrail vs. NonMetS-Nonfrail *p* = 0.04 and MetS-Nonfrail vs. NonMetS-frail *p* = 0.00003), systolic blood pressure (MetS-Nonfrail vs. NonMetS-Nonfrail *p* = 0.002), fasting glucose concentration (MetS-Nonfrail vs. NonMetS-Nonfrail *p* = 0.02 and MetS-Nonfrail vs. NonMetS-frail *p* = 0.001), total cholesterol (MetS-Nonfrail vs. MetS-frail *p* = 0.03), and HDL cholesterol (MetS-Nonfrail vs. MetS-frail *p* = 0.02). The differences may be due to the definition of the metabolic syndrome. 

NonMetS-Nonfrail individuals had the best HDL cholesterol level compared to the other groups and post hoc analysis showed the highest difference with NonMetS-frail group (*p* = 0.002) and MetS-frail elderly patients (*p* = 0.0001).

Overall, the data suggest that MetS individuals, both frail and nonfrail, have higher risk factors for cardiovascular disease compared to NonMetS individuals.

Measurement of CBC revealed that NonMetS-frail elderly persons had the highest white blood cells count (however, within reference range) in comparison with other elderly groups (*p* = 0.0146), which may suggest an inflammatory state not as prominent in the other groups. MetS-frail individuals exhibited significant deficits in red blood cell count (RBC), hemoglobin levels (HGB), and hematocrit (HCT), indicating anemia or worse overall nutritional status compared to NonMetS groups.

A study examining the correlations between SOD-1 activity and various metabolic are presented in Table 2. Significant results (at *p* < 0.05) are highlighted in gray. 

In group NonMetS-Nonfrail, SOD-1 was negatively correlated with age, meaning the older the age, the lower the SOD-1 (r = −0.6293; *p* < 0.05), and positively correlated with TC (r = 0.4866, *p* < 0.05) and LDL-C (r = 0.5160, *p* < 0.05). In group NonMetS-frail, SOD-1 was negatively correlated with HDL-C (r = −0.4586; *p* < 0.05) and with PLT (r = −0.4650; *p* < 0.05). In group MetS-Nonfrail, SOD-1 strongly correlated with blood hematocrite (r = 0.8829; *p* < 0.05). In group MetS-frail, SOD-1 was strongly negatively correlated with fasting glucose (r = −0.8426, *p* < 0.05). The findings suggest that SOD-1 levels may be influenced by age, total cholesterol, HDL cholesterol, and fasting glucose levels in certain groups of seniors.

## 4. Discussion

The SOD-1 is a crucial antioxidant enzyme in humans that regulates the amount of superoxide anion in the cytosol—essential for normal cellular metabolism (regulating signaling pathways and combating pathogens)—but it also arises under the influence of pathological processes and external factors, limiting the potential formation of hydroxyl radicals from •O2^−^ [38]. With SOD-1, cells transform superoxide anion radicals into oxygen and hydrogen peroxide.

Decreased activity of the enzyme could lead to the build-up of hydrogen peroxide, potentially contributing to cellular aging [39]. In our NonMetS-Nonfrail group, SOD-1 activity was negatively correlated with age, indicating that older individuals may have lower SOD-1 activity, potentially reflecting increased oxidative stress or reduced antioxidant defenses. This correlation was not observed in other groups. However, our elderly investigated groups had the same activity of SOD-1. The NonMetS-frail was the oldest age group in comparison with metabolic syndrome groups; this suggests frailty is associated with an older demographic. Additionally, there were higher WBC counts, indicating the importance of inflammation in this group. What is more, the MetS-frail group had the worst CBC results in comparison with other groups. This suggests frailty together with the metabolic factor, which does not influence SOD-1 activity, may worsen hematological results. Thus, in frail individuals there is an increased risk of adverse health outcomes due to inflammation and/or anemia.

Excess O_2_ production is seen in individuals who use to use ethanol and/or smoke. However, research examining the activity of SOD-1 in erythrocytes has produced varying outcomes: Some studies found increased activity in individuals consuming alcohol/smokers, while others reported reduced levels in alcoholics, and a few observed no significant differences at all [40,41,42]. Thus, to avoid interpretation problems with the results of SOD-1 activity, in our study, we only examined individuals who did not consume alcohol nor smoke.

The result of enzyme activity is not solely determined by the gene structure related to the quantity of the protein and its enzymatic role. Recently, we have gained insight into epigenetic regulation, which encompasses changes in gene expression that do not involve DNA sequence [43]. Epigenetics explains the phenotypic variations that occur due to varying levels of gene expression in genetically identical cells [44,45].

To maintain the enzyme’s full activity, Zn^2+^ ions are required and stabilize SOD-1, whereas Cu^2+^ ions are crucial for its catalytic (red-ox) activity [46]. It was observed that there was reduced SOD-1 activity and zinc concentration in obese men compared to a group of healthy, normal BMI persons [47]. There is evidence to suggest that SOD-1 activity may be affected by metabolic syndrome in elderly individuals [34]. Metabolic syndrome is a cluster of conditions that includes excess body fat around the waist, high blood pressure, elevated blood sugar, and abnormal cholesterol levels. Waist circumference in MetS-Nonfrail was significantly increased compared to all NonMetS individuals (both Nonfrail and frail). This indicates that MetS-Nonfrail individuals have a concerning waist circumference, which is a key indicator of increased cardiovascular risk and metabolic dysfunction. Additionally, MetS-Nonfrail individuals exhibit the highest median of SBP, which is significantly greater than that of NonMetS-Nonfrail and NonMetS-frail, presenting an important cardiovascular risk factor. It is associated with chronic low-grade inflammation and oxidative stress, which can impact the activity of antioxidant enzymes such as SOD-1. However, in our elderly patients we did not find significant differences of SOD-1 among elderly metabolic and non-metabolic syndrome populations. But, in those with metabolic syndrome who also were frail, the SOD-1 activity adversely corresponded with fasting glucose, suggesting the role of declining antioxidant capacity in the multiple syndromes. This indicates that lower SOD-1 activity might be associated with poorer glycemic control or increased oxidative stress in frail individuals with MetS. Our MetS elderly groups exceed the glucose levels in comparison with NonMetS individuals, highlighting metabolic dysregulation. However, HbA1c levels did not show significant differences across groups, suggesting that overall glycemic control might be similar. Furthermore, researchers found that patients with diabetes exhibited greater superoxide dismutase activity in erythrocytes compared to individuals without diabetes (including those with normal and abnormal fasting glucose levels) [48]. Yet, it was also found, in non-alcoholic fatty liver disease (NAFLD) with and without diabetes mellitus, SOD-1 activity was decreased [49].

Additionally, maintaining optimal activity of SOD-1 by staying active, adopting a nutritious diet, and incorporating other healthy habits may lower the chance of developing metabolic syndrome and slow down frailty [50]. Grygiel et al. discovered a link between high HDL-C levels and increased SOD-1 activity among postmenopausal obese women [51]. In our investigated NonMetS-Nonfrail elderly patients, there was only an age and SOD-1 association, while in those with metabolic syndrome and/or frailty, SOD-1 was related to metabolic parameters. Furthermore, in those with frailty syndrome, SOD-1 was inversely related to HDL cholesterol.

Dyslipidemia was found to play a role in the generation of oxidative stress in senior individuals, leading to reduced levels of HDL cholesterol [52]. Our NonMetS individuals, particularly the nonfrail, demonstrated the best lipid profiles. The HDL particles have a beneficial effect against atherosclerosis as well as the capacity to induce insulin secretion [53]. While HDL and SOD-1 serve distinct functions, they intersect in the context of cardiovascular health and oxidative stress. By limiting oxidative stress, HDL helps maintain the functional integrity of endothelial cells, contributing to vascular health. Additionally, there are inverse relationships between SOD-1 activity and various cardiovascular markers such as pressure wave velocity, augmentation index, arterial stiffness index, pulse pressure, and HDL cholesterol levels [54]. In conditions of elevated oxidative stress, such as metabolic syndrome, HDL may become modified by oxidation, impacting its functionality. SOD-1 can help mitigate inflammation and oxidative damage to HDL, thus preserving its role in reverse cholesterol transport. What is more, HDL’s antioxidative properties may be enhanced by the presence of SOD-1 [52]. Our elderly frail and metabolic syndrome patients had dramatically low HDL cholesterol levels. Meanwhile, in the frail but NonMetS group, SOD-1 was negatively correlated with TC and with HDL-C. Furthermore, the correlation between fasting glucose and SOD-1 was highly negative, indicating that metabolic factors are related with antioxidants reduction.

Regarding SOD, the results of this study suggest that even within the same frailty category, changes in factors related to metabolic syndrome can lead to opposite outcomes. A similar phenomenon has been reported in cancer patients, who, in some cases, may be also frail [55,56]. Cancer cells are characterized by elevated levels of reactive oxygen species (ROS), which heightens their reliance on the antioxidant defense system. Consequently, SOD1 is often overproduced in various types of cancers. The SOD-1 concentration was shown to predict overall cancer survival; however, the SOD activity in cancer has shown contradictory results [57]. Comparing with our study, in frailty and/or metabolic syndrome elderly persons, the varying relationships between SOD-1 activity and different clinical parameters suggest that oxidative stress may influence various health outcomes. Further studies should investigate how antioxidant defense systems affect frailty and metabolic health. Incorporating measures of oxidative stress, inflammatory markers, and detailed metabolic profiles in clinical assessments could provide a more comprehensive understanding of an elderly individual’s health status.

### Strengths and Limitations of the Study

With a growing elderly population globally, it is increasingly important to address health concerns related to aging, particularly the connections between antioxidant defense, metabolic syndrome, and frailty in older adults. Our findings stress the importance of monitoring metabolic parameters alongside frailty assessments in clinical settings to mitigate health risks for aging populations. However, the limited number of individuals in this study with both metabolic syndrome and frailty may reduce the statistical power of the findings and hinder the ability to generalize the results to the wider elderly population. Longitudinal studies may be necessary to determine whether changes in SOD-1 activity and metabolic factors truly affect one another over time. What is more, measurement of related cofactors that may influence SOD-1 activity, such as anemia, vitamin deficiency, or trace elements of Cu and Zn, are needed. Research using genomics and proteomics may also be useful.

## 5. Conclusions

The results of this study highlight the intricate relationship between superoxide dismutase 1, metabolic syndrome, and/or frailty syndrome in the elderly population. Specifically, our results demonstrate that aging is accompanied by a significant decrease in the activity of the antioxidant enzyme superoxide dismutase-1 (SOD-1), with variations observed depending on the presence of metabolic syndrome and/or frailty syndrome. In particular, the correlation between SOD-1 activity and fasting glucose levels in frail individuals highlights how metabolic dysregulation may exacerbate oxidative stress, potentially accelerating the aging process and contributing to the development of chronic conditions. What is more, the inverse relationship between SOD-1 activity and critical metabolic parameters such as total cholesterol and HDL-C raises important questions about the efficacy of existing therapeutic strategies to manage these conditions in the elderly.

Understanding the underlying mechanisms that lead to decreased SOD-1 activity and how these might be influenced by lifestyle factors (such as diet and physical activity) could open new avenues for preventive health strategies aimed at maintaining antioxidant defenses in older adults. Further research should focus on exploring the therapeutic potential of enhancing SOD-1 activity in this vulnerable population. By tackling both metabolic and oxidative stress challenges, we may promote healthier aging, enhance the quality of life for older adults, and, ultimately, reduce healthcare costs.

## Figures and Tables

**Table 1:** Characteristics of the studied groups.

Variable	NonMetS-Nonfrail n = 19	NonMetS-Frail n = 20	MetS-Nonfrail n = 17	MetS-Frail n = 10	*p*
Age [years]	70.0 (66.0–75.0)	81.0 (73.0–88.5)	71.0 (68.0–74.0)	71.5 (68.0–78.0)	0.004
Waist [cm]	83.0 (78.0–92.0)	80.0 (71.0–86.5)	95.0 (92.0–102.0)	80.0 (74.0–98.0)	0.0001
SBP [mmHg]	130.0 (125.0–140.0)	122.5 (107.5–140.0)	140.0 (135.0–150.0)	142.5 (140.0–145.0)	0.001
DBP [mmHg]	80.0 (70.0–90.0)	77.5 (67.5–80.0)	85.0(80.0–85.0)	77.5 (70.0–90.0)	>0.05
G 0’ [mg/dL]	93.0 (90.4–97.5)	89.1 (86.0–101.0)	111.0 (103.2–115.2)	125.2 (114.8–149.0)	0.0000
Mean G [mg/dL]	117.0 (111.0–123.0)	117.0 (108.0–131.0)	123.0 (114.0–131.0)	126.0 (108.0–126.0)	>0.05
HbA_1c_ [%]	5.7 (5.5–5.9)	5.7 (5.4–6.2)	5.9 (5.6–6.2)	6.0 (5.4–6.0)	>0.05
TC [mg/dL]	200.0 (185.0–222.0)	164.5 (125.5–184.0)	186.0 (160.0–201.0)	133.5 (116.0–151.0)	0.0000
TG [mg/dL]	82.0 (70.0–120.0)	95.0 (70.0–133.0)	92.0 (70.0–182.0)	148.5 (116.0–188.0)	>0.05
HDL-C [mg/dL]	66.7 (59.2–71.4)	45.5 (39.0–50.8)	54.0 (45.0–65.8)	27.0 (24.1–37.0)	0.0000
LDL-C [mg/dL]	116.0 (99.6–134.8)	78.0 (62.6–113.2)	107.6 (90.0–118.7)	74.4 (70.0–88.0)	0.003
WBC [G/L]	5.4 (4.9–6.9)	7.8 (5.9–9.4)	6.0 (5.3–7.5)	7.2 (6.5–9.2)	0.0146
PLT [G/L]	303 (273–318)	324 (246–399)	242 (164–338)	402 (239–482)	>0.05
RBC [T/L]	4.56 (4.36–4.80)	4.29 (4.09–4.61)	4.77 (4.52–5.38)	4.04 (3.90–4.69)	0.0453
HCT [%]	40.5 (38,8–41,8)	37.0 (33.4–40.2)	41.9 (41.0–42.1)	36.2 (33.0–40.8)	0.0081
HGB [g/dL]	12.7 (12.1–13.8)	11.6 (11.1–12.6)	12.8 (12.3–14.2)	12.1 (11.0–13.9)	0.0197
SOD-1 [U/gHGB]	1202.8 (1002.6–1622.7)	1067.2 (759.1–1496.5)	919.8 (809.7–1166.6)	1270.3 (1121.0–1407.4)	>0.05

SBP—systolic blood pressure, DBP—diastolic blood pressure, G0’—fasting glucose, Mean G—mean glucose concentration, HbA_1c_—glycated hemoglobin, TC—total cholesterol, TG—triacylglycerols, HDL-C—high-density lipoproteins cholesterol, LDL-C—low-density lipoproteins cholesterol, WBC—white blood cells, PLT—platelets, RBC—red blood cells, HCT—hematocrite, HGB—hemoglobin, SOD-1—superoxide dismutase.

**Table 2:** The correlations between SOD-1 activity and clinical and biochemical parameters in the studied elderly groups; all significant correlations are highlighted (at *p* < 0.05).

Variable	NonMetS-Nonfrail SOD-1 Activity Spearman’s R Coefficient	NonMetS-Frail SOD-1 Activity Spearman’s R Coefficient	MetS-Nonfrail SOD-1 Activity Spearman’s R Coefficient	MetS-Frail SOD-1 Activity Spearman’s R Coefficient
Age	−0.6293	0.0023	0.322-	0.6000
Waist	−0.0079	−0.3003	0.0602	−0.1471
SBP	0.1348	−0.1881	0.0625	0.0294
DBP	0.3978	−0.0927	−0.2555	0.1852
G 0’	−0.2511	0.2339	−0.0686	−0.8426
HbA_1c_	0.2941	0.0818	0.0012	0.6377
TC	0.4866	−0.4301	0.1669	−0.6000
TG	0.1795	−0.1343	−0.3514	−0.2000
HDL-C	0.0246	−0.4586	0.0245	0.3143
LDL-C	0.5160	−0.2075	0.3211	−0.6000
WBC	−0.3426	−0.2250	0.2143	0.4857
PLT	0.1366	−0.4650	0.2143	0.1428
RBC	−0.5315	0.1714	0.6071	−0.2571
HCT	−0.5315	−0.1023	0.8829	−0.2571
HGB	−0.2827	−0.0956	0.4196	−0.0857

SBP—systolic blood pressure, DBP—diastolic blood pressure, G0’—fasting glucose, HbA_1c_—glycated hemoglobin, TC—total cholesterol, TG—triacylglycerols, HDL-C—high-density lipoproteins cholesterol, LDL-C—low-density lipoproteins cholesterol, WBC—white blood cells, PLT—platelets, RBC—red blood cells, HCT—hematocrite, HGB—hemoglobin, SOD-1—superoxide dismutase.

## Data Availability

Data is contained within the article.
